# Fevers

**Published:** 1905-09-16

**Authors:** 


### Progress in Medicine and Surgery.

### FEVERS.

**Epidemic Cerebro-spinal Meningitis.**—Cerebro-spinal meningitis was first described in 1805, by Vieussieu of Geneva, who studied an epidemic in his city. Next year, in 1806, its first appearance in America was recorded, and since that time various outbreaks have been chronicled in the latter country and in Europe, while recent severe epidemics in Germany and in the United States have aroused public anxiety as well as stimulated professional interest. Since 1860 the disease appears to have taken the form of occasional virulent outbreaks, linked by scattered sporadic cases. The exact ætiological conditions which favour the existence of these sporadic cases and lead to the occasional graver outbreaks are not yet clearly known. But certain predisposing circumstances have been observed. Thus the vast majority of cases occur among children and young adults, and although all classes of people and even animals, domestic and wild, have been affected, poor food, overcrowding, insanitary surroundings, over-fatigue, and mental worry predispose to this as to so many infective diseases. The seasonal occurrence, in winter and in spring, coincides with that of pneumonia with epidemics of which disease it sometimes concurs. Infection from one patient to another has been said to take place in about one case in ten,[1] but it is exceptional to find more than one patient in a house, although multiple cases have been recorded in Norway, in Ireland by Reford [2] and by Osler who described five cases in one family. In some instances a place infection seems to prevail. Thus Netter recalls that in an outbreak in Fiji successive batches of workmen despatched to one particular island became infected. Neisser has shown that the infective organism is readily transported by air currents, and Germano has drawn attention to the length of time—90 days—during which it resists desiccation.[3] From the proximity of the nasal cavities to the brain and its meninges entrance of the germ by the upper respiratory tract has been suspected. Thus Jacobi has for some years advocated the prophylactic use of antiseptic nasal sprays. Weigert, Strümpell, Jaeger, Netter and others also believe that the pathogenic organism penetrates to the cerebral membranes through the ethmoidal plate, and Busset has made three series of interesting experiments in which he has produced the disease in guinea-pigs and rabbits by introducing into their nasal fossæ probes charged with the nasal mucus or the cerebro-spinal fluid of patients suffering from the complaint. In some epidemics a preliminary nasal obstruction or catarrh has been noticed in as many as one-fourth [4] of the cases. The organism has often been found in the nasal mucus of patients,[5] in 18 cases examined by Scherrer. On the other hand, Schiff has found it in the nasal mucus of the healthy, and one is thus reminded of the fact that the upper respiratory tract is not infrequently the habitat of the Klebs-Loeffler bacillus in the apparently healthy as well as in the victims of diphtheria. It is interesting also to note that epidemics of "pink eye" [6] in horses sometimes coincide with outbreaks of meningitis in human subjects, and in one case an attack of "pink eye" in a child was followed by an attack of epidemic meningitis.[7] Entrance in this case may have been effected through the conjunctival sac. Since Weichselbaum isolated the diplococcus intracellularis in 1887 this organism has been generally accepted as the commonest specific agent, and it is found in the cerebro-spinal fluid of the majority of patients. That it is not the sole cause, however, as has been maintained by some bacteriologists, seems clear from the facts emphasised by Netter in the article already quoted. He points out that while the diplococcus alone has been found in certain epidemics, in other no less typical outbreaks of meningitis, the pneumococcus, or the modified pneumococcus of Bonôme, has been found. Further Netter asserts that he has "constantly seen parallel cases due to the pneumococcus and to the organism of Weichselbaum," and also that "it is possible to affirm, not only the existence of epidemics of cerebro-spinal meningitis with pneumococci and with the micrococcus of Weichselbaum, but also the coexistence in the same epidemic of cases caused by one or the other of these microbes." The close epidemiological relationship between epidemic meningitis and pneumonia may be recalled here. The apparently dual bacteriology is interesting, but perplexing. An explanation has been sought in a suggested close relationship between the two micrococci, and possibly some recent work done by Maher [8] in America may help to solve the question. He has obtained cultural and inoculation results which suggest the view that both diplococci and pneumococci are phases in the life cycle of a larger rod-shaped organism or a yeast-like organism. Of special symptoms, and the symptomology is very varied, headache and Kernig's sign are perhaps of most diagnostic value. Pain in the head extending to the neck and back is a very constant feature, lasting as long as consciousness is maintained. Kernig's sign is also very constant, and may be regarded as almost, though not quite, pathognomonic of meningeal inflammations. Another early pressure sign has been noted by Speer,[9] namely, a tendency for one or both feet to turn inwards until they cross one another. In diagnosis lumbar puncture is invaluable and free from risk if done with ordinary care. The presence of the specific micro-organisms can thus often be determined and a cytological examination of the centrifugalised fluid made. Too much stress must not, however, be laid on the fact, useful for differential diagnosis, that in the epidemic form the polymorphonuclear neutrophiles are immensely increased, while in meningitis associated with tubercle the exudate consists almost entirely of lymphocytes, for in the later stages of the illness lymphocytes may almost entirely replace the multinuclear leucocytes so abundant in the earlier effusion. Netter lays more stress for diagnosis on the macroscopic appearance of the lymph clot, which in the epidemic disease is yellowish, soft, and readily spread out, while in tubercular meningitis it is white, resistant, and spreads out with difficulty. Although well established as a safe and certain diagnostic measure, the therapeutic worth of lumbar puncture is less recognised and as a method of cure it is still on its trial. There is, however, an increasing inclination to regard it as a hopeful procedure. Simple withdrawal of the fluid is more often practised, but in some cases the injection of antiseptic solutions into the spinal canal after withdrawal is attempted and with some measure of success. Lysol in one per cent. solution is a favourite application and about 10 cc. may be injected at once. Lenhartz[10] advocates early and repeated puncture, and claims success for the treatment. He withdraws from 30 to 50 cc., and repeats the process as often as pressure symptoms recur. The best spot for puncture is the junction of a perpendicular dropped from the iliac crest with the line of the spine, the patient lying on one side with the thighs strongly flexed. The fluid should always be withdrawn slowly and it is advisable to measure the intraspinal pressure. Relief of pressure symptoms for the time almost always follows, even in cases not permanently benefited. The withdrawal of purulent fluid removes a large number of pathogenic organisms and unless the foramen of Magendie is closed it also relieves ventricular dropsy, and hence may lessen the occurrence of a serious sequela—chronic hydrocephalus. Certainly where the pus is present under considerable tension it seems reasonable to expect a relief of constitutional and local symptoms such as one observes on the evacuation of pus from other regions of the body, as *e.g.* from a subcutaneous abscess. The application of hydrotherapy was introduced by Aufrecht in 1894 and has received the approval of the majority of those who have employed it, but the method of application has varied considerably. Thus Aufrecht gave only one bath daily, while Barr employed a prolonged immersion of eleven days or more. Netter, who has had extensive experience of, and warmly recommends, the treatment, advises a hot bath lasting from half to three-quarters of an hour, repeated four or five times a day. Recent and enthusiastic testimony as to the benefit of this treatment comes from Russia, where Rojansky[11] has employed it for five years in the treatment of 51 patients in his women's wards. His method is to give a bath at a temperature of 104° F. for 15 or 20 minutes once or twice a day, applying an ice cap to the head at the same time. Pain, restlessness, delirium, and unconsciousness are rapidly and decidedly relieved. Of the cases so treated 17 died, a mortality of 33 per cent. Rojansky's results are a valuable contribution to the effect of the bath treatment, because an accurate control was provided by the simultaneous treatment, without baths, of 50 cases admitted to the male wards, of whom 40, or 80 per cent., died. An interesting contribution to this subject has been made by the employment, in a few cases, of subcutaneous injections of diphtheria antitoxin. Wolff[12] of Hartford, Conn., was led to use it in three or four cases, which recovered, as a result of his observation that a pronounced antagonism existed between the diplococcus intracellularis and the Klebs-Loeffler bacillus, and that pure cultures of the former were killed by the addition of antidiphtheritic serum. Large doses, 6,000 to 10,000 units were employed. Waitzfelder, of New York, has tried it also and reports a few striking cases of recovery, but although the treatment seems deserving of further trial no certain conclusions can be deduced from the small number of cases hitherto treated. It is difficult readily to assess the value of any particular form of treatment as epidemics vary much in severity, and while the mortality rarely falls as low as 30 it has reached as high as 77 per cent. Moreover epidemics are usually somewhat equably distributed in the area affected so that it is not the rule for any very large number of patients to come under the treatment of one physician. Hence statistics showing the result of treatment must be accepted with perhaps more than average caution.

[1] Med. Rec., May 6, p. 716. [2] Lancet, May 6, p. 1214. [3] Med. Rec., April 15, p. 562. [4] Ibid, May 6, p. 716. [5] Lancet, April 15. [6] Med. Rec., March 11, p. 365. [7] Ibid, Feb. 4, p. 195. [8] Ibid, May 6. [9] Ibid, April 15. [10] Lancet, April 15, p. 1011. [11] Med. Rec., April 1. [12] Ibid, March 11.

